# Growth and productivity assessments of peanut under different irrigation water management practices using CSM-CROPGRO-Peanut model in Eastern Mediterranean of Turkey

**DOI:** 10.1007/s11356-021-17722-w

**Published:** 2021-12-03

**Authors:** Semih Metin Sezen, Ishfaq Ahmad, Muhammad Habib-ur-Rahman, Ebrahim Amiri, Servet Tekin, Kadir Can Oz, Clever Mwika Maambo

**Affiliations:** 1 Department of Irrigation and Agricultural Structures, Faculty of Agriculture, Cukurova University, 01330 Adana, Turkey; 2 Resilient Agriculture Department, Asian Disaster Preparedness Center (ADPC), Bangladesh, Pakistan; 3 Institute of Crop Science and Resource Conservation (INRES), Crop Science Group, Rheinische Friedrich-Wilhelms-Universität Bonn, Katzenburgweg 5, 53115 Bonn, Germany; 4 MNS-University of Agriculture, Multan, Pakistan; 5 Water Engineering Department, Islamic Azad University, Lahijan, Iran; 6 Department of Biosystems Engineering, Faculty of Agriculture, Kahramanmaras University, 46100 Kahramanmaras, Turkey

**Keywords:** Crop simulation, Model evaluation, Water stress, Irrigation strategies, Decision-making

## Abstract

Irrigation water scheduling is crucial to make the most efficient use of ever-decreasing water. As excessive irrigation decreases yield, while imprecise application also causes various environmental issues. Therefore, efficient management of irrigation frequency and irrigation level is necessary to sustain productivity under limited water conditions. The objective of the current study is to assess the water productivity at various irrigation regimes during peanut crop growing seasons (2014 and 2015) in Eastern Mediterranean, Turkey. The field experiments were conducted with treatments consisting of three irrigation frequencies (IF) (IF_1_: 25 mm; IF_2_: 50 mm; and IF_3_: 75 mm of cumulative pan evaporation (CPE)), and four irrigation water levels (WL_1_ = 0.50, WL_2_ = 0.75, WL_3_ = 1.0, and WL_4_ = 1.25). WL_1_, WL_2_, WL_3_, and WL_4_ treatments received 50, 75, 100, and 125 of cumulative pan evaporation. The CSM-CROPGRO-Peanut model was calibrated with experimental data in 2014 and evaluated with second-year experimental data (2015). The model simulated seed yield and final biomass (dry matter) reasonably well with low normalized root mean square error (RMSE_*n*_) in various irrigation intervals. The model simulated reasonably well for days to anthesis (RMSE = 2.53, *d*-stat = 0.96, and *r*^2^ = 0.90), days to physiological maturity (RMSE = 2.55), seed yield (RMSE = 1504), and tops biomass dry weight at maturity (RMSE = 3716). Simulation results indicated good agreement between measured and simulated soil water content (SWC) with low RMSE_*n*_ values (4.0 to 16.8% in 2014 and 4.3 to 18.2% in 2015). Further results showed that IF_2_I_125_ irrigation regime produced the highest seed yield. Generally, model evaluation performed reasonably well for all studied parameters with both years’ experimental data. Results also showed that the crop model would be a precision agriculture tool for the extrapolation of the allocation of irrigation water resources and decision management under current and future climate.

## Highlights

- DSSAT-CROPGRO-Peanut model was used to optimize irrigation scheduling for drip irrigated peanut.
- Twelve irrigation strategies were evaluated with the model.
- The genetic coefficients of peanut used in the model have been determined.
- CROPGRO-Peanut model satisfactorily simulated peanut yield and phenology.
- CROPGRO was evaluated for predicting peanut yield, SWC, LAI, ET, WP, and IWP.

## Introduction

Peanut (*Arachis hypogaea* L.) is a major industrial and food crop in Turkey as in many parts of the world (Sezen et al. 2019). Peanut crop production  was 0.40 tons ha^−1^ in 2019, and 86.6% of peanut production was from East Mediterranean part of Turkey (TUIK 2020). The average number of world annual peanut production was 45.9 million tons in 2018. Data from the Food and Agriculture Organization revealed that China, India, and Nigeria are the world’s three major peanut-producing countries. While Turkey produces 0.38% of world peanut production (FAOSTAT 2020).

Water resources are depleting over the world (Hashemi et al. 2020; Meena et al. 2019; Saddique et al. 2020c), and less irrigation water will be available for crop production. Therefore, the management and judicious use of irrigation water is a challenge (Saddique et al. 2020a). As, water is essential for successful crop production, therefore, any shortage and poor management have negative impacts on crop yield (Magombeyi et al. 2018; Saddique et al. 2020b). The farmers in the East Mediterranean part of Turkey commonly used wild flooding, furrow, and basin, resulting in high water losses and low irrigation efficiencies which create drainage and salinity problems other than irrigation water losses (Tekinel et al. 1989; Shafqat et al. 2019). The application of water according to crop requirement increases productivity and minimizes water loss in the form of runoff and percolation (Nikolaou et al. 2020). Traditional surface irrigation methods are used to irrigate peanut crop in East Mediterranean region of Turkey; farmers apply the water through flood without considering the actual consumptive requirements of the peanut that reduced the water efficiency, and also reduce the crop yield. Supplemental irrigation during water stress is critical to ensure and improve plant development and produce high yield and top-quality peanut in the Southeastern part of Mediterranean (Sezen et al. 2019). The appropriate irrigation scheduling is useful to minimize water use, increase water productivity, and save economic returns while maintaining production (Fereres and Soriano 2007; Rahman et al. 2016, 2021; Shafqat et al. 2021). Additionally, irrigation frequency (IF) and irrigation level are some of the most prominent factors in drip irrigation management due to the soil water pattern and water percolation under the root zone (Wang et al. 2006). There are limited studies that have been focused to improve irrigation water use efficiency and reduce water losses besides improving the peanut crop yield. There is a need to maximize the production per unit of water consumed to remain economically competitive and to sustain irrigated agriculture.

Different precision tools are being used for irrigation water management and decision management under water-scarce scenarios in the world. Computer simulation models, such as Decision Support System for Agro-technology Transfer (DSSAT) (Hoogenboom et al. 2010), have the potential to develop irrigation scheduling and to evaluate the impact of water stress on plant growth and development, as these crop simulations consider the soil–plant-atmosphere complex interaction. The CSM-CROPGRO module under DSSAT has been successfully applied for irrigation management, particularly cumulative water stress during the growing season (Wajid et al. 2013). The CSM-CROPGRO Peanut is a process-based, management-oriented model that can simulate the growth and development of peanut as affected by varying levels of water and irrigation intervals (Boote et al. 1998a, b). Previously, the model which has been successfully applied and used for decision support for different crop management under different contrasting environments showed the ability and potential (Wajid et al. 2014; Amin et al. 2017; Rahman et al. 2019, 2020).

The present study aims to manage the irrigation water through deficit irrigation using a decision support system. The goals of the current study were to evaluate growth (leaf area index, biomass), phenology, evapotranspiration, seed and biological yield, water, and irrigation productivity under full and deficit irrigation practices using field data and model approach. The main objective of this study is to adapt and evaluate the CSM-CROPGRO-peanut model to simulate the growth, development, and yield in response to irrigation interval and levels in Eastern Mediterranean of Turkey for efficient use of irrigation water.

## Materials and methods

### Environmental conditions of the study site

**Fig. 1:**
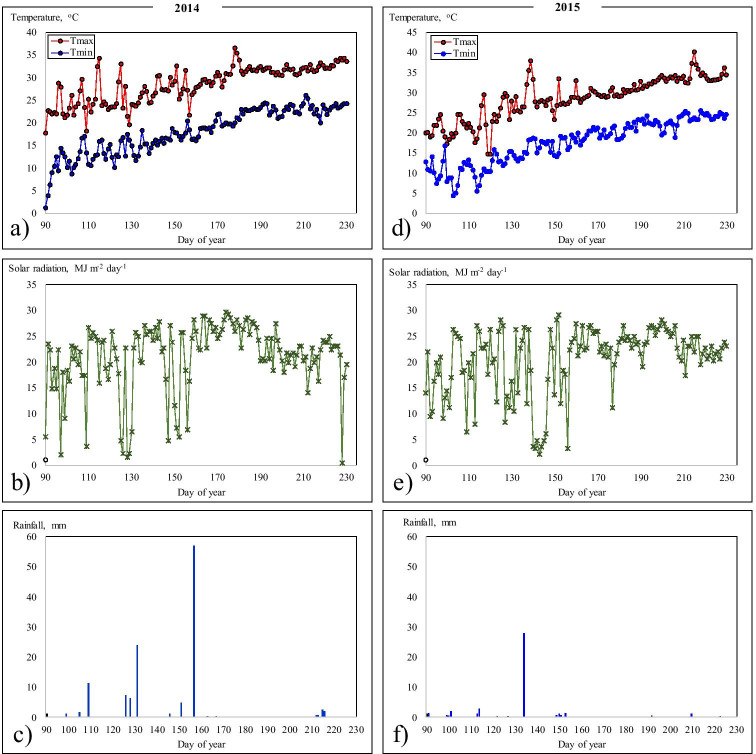
Daily maximum and minimum air temperatures, solar radiation, and rainfall during peanut crop growing seasons of 2014 and 2015

The experimental site is located in the Eastern Mediterranean of Turkey, and 2 years (2014 and 2015) of experiments were conducted at the Soil and Water Resources Unit of Alata Horticultural Research Station, Tarsus, Turkey (37° 01′ N latitude and 35° 01′ E longitude; 60 m above mean sea level). Daily maximum and minimum temperature, rainfall, and solar radiation for the cropping period were recorded with an automatic weather station installed in the experimental field and used as input data for the model. The maximum and minimum temperatures varied in the ranges 27.9–37.7 °C and 1.9–20.0 °C, respectively, during the 2014 growing season, with a total rainfall of 223 mm. While, during the 2015 cropping season, a total of 103-mm rainfall was received, with maximum and minimum temperatures in the ranges 29.5–40.4 °C and 4.5–18.9 °C, respectively, as shown in Fig. 1. The collected weather data were used to create a weather module of the model.

The values of the measured field water capacity at the site varied between 29.46 and 32.08 g g^−1^, and wilting point increased from 15.81 to 19.83 g g^−1^ on a dry weight basis. The soil contained high ratios of sand (32.5–43.6%) followed by clay (28.5–37.0%) and silt (27.9–32.6%) and it could be categorized as clay-loam. The dry soil bulk densities ranged from 1.38 to 1.58 g cm^−3^ along with 0.90-m profiles. The available water in the 0.90 m of soil depth was computed 155 mm. The soil had a pH of 7.5–7.8, organic matter content of 0.53–1.43%, total nitrogen, and phosphorus of 0.04%, while potassium was 0.42% (Sezen et al. 2017). Soil physical and chemical characteristics for each soil layer were then implemented to create a soil module in the model.

### Experimental detail

Crop experiments consisted of twelve treatment combinations, including three irrigation frequencies (IF) (IF_1_: 25 mm; IF_2_: 50 mm; IF_3_: 75 mm of cumulative pan evaporation (CPE)) and four irrigation water levels (WL_1_ = 0.50, WL_2_ = 0.75, WL_3_ = 1.00, and WL_4_ = 1.25). While, WL_1_, WL_2_, WL_3,_ and WL_4_ treatments received 50, 75, 100, and 125 of CPE respectively. Treatment combinations were used and replicated four times. The experiment was designed in a split-plot arrangement (main factor: irrigation frequency; sub factor: irrigation water level). Peanut seeds of cultivar NC-7 (Virginia-type peanut) were planted on May 20, 2014, and May 08, 2015. Compound fertilizer (18% N, 46% P_2_O_5_, and 0% K_2_O) was used at a rate of 200 kg ha^−1^ during sowing. While the remaining N (400 kg ha^−1^) was applied as ammonium nitrate (33% N) during the flowering growth stage during both years. The harvest was carried out by manually collecting 6 m portions of three adjacent central rows at each subplot on November 06, 2014, and October 09, 2015. The data of phenology (days to anthesis, maturity), growth (leaf area index and biomass), and yield were collected from the experiments, which were used to calibrate and evaluate the model.

### Calibration and evaluation of CSM-CROPGRO model

The model (CSM-CROPGRO-Peanut model V 4.7.5) was calibrated with field experimental data of 2014 and evaluated with second-year field data (2015). The parameters used for model parameterization were phenology (days to anthesis, and maturity), leaf area index (LAI), and biological (dry biomass) and grain yield. The genetic coefficients for peanut cultivar NC-7 were estimated using the generalized likelihood uncertainty estimation (GLUE) approach; similar methods were previously used for CROPGRO-model calibration as Ahmed et al. (2018); Rahman et al. (2018); Vanli et al. (2019).

The accuracy of the model was determined by comparing the simulated values with observed by using statistical indices such as coefficient of determination (*r*^2^), the root mean square error (RMSE) (Wallach and Goffinet 1987) and the index of agreement (*d*) (Willmott et al. 1985), and normalized root mean square error (RMSE_*n*_) as shown in Eqs. 1–5. The values of RMSE and *d* indicate the degree of agreement between the predicted values with their corresponding observed values, and a low RMSE value and *d* value approaching unity are desirable (Dangthaisong et al. 2006). The RMSE and RMSE_*n*_ were computed using the following equations:

1 $${r}^{2}=\frac{{\left(\left(\sum_{i=1}^{n}\left({O}_{i}-{\overline{P} }_{i}\right)({\widehat{P}}_{i}-{\overline{P} }_{i}\right))\right)}^{2}}{\sum_{i=1}^{n}{\left({O}_{i}-\overline{O }\right)}^{2}\sum_{i=1}^{n}{\left({\widehat{P}}_{i}-{\overline{P} }_{i}\right)}^{2}}$$

2 $$\mathrm{RMSE}=\sqrt{\frac{\sum_{i=1}^{n}{\left({P}_{i}-{O}_{i}\right)}^{2}}{n}}$$

3 $${\mathrm{RMSE}}_{n}=\frac{100{\left[\sum_{i=1}^{n}\frac{{({P}_{i}-{O}_{i})}^{2}}{n}\right]}^{0.5}}{\overline{O} }$$

where *n* is the number of observations, *P*_*i*_ is the predicted value for the *i*th measurement and *O*_*i*_ is the observed value for the *i*th measurement. The *d*-statistic (*d*-stat) or “index of agreement” value was calculated as follows;

4 $$d=1-\left|\frac{\sum_{i=1}^n{\left({P}_i-{O}_i\right)}^2}{\sum_{i=1}^n{\left(\left|{\overset{\acute{\mkern6mu}}{P}}_i\right|+\left|\overset{\acute{\mkern6mu}}{O_i}\right|\right)}^2}\right|,\kern0.5em 0\le d\le 1$$

where *n* = number of observations, *P*_*i*_ = predicted value for the *i*th measurement, *O*_*i*_ = observed value for the *i*th measurement, Ō = the overall mean of observed values, *P’*_*i*_ = *P*_*i*_– Ō, and *O’*_*i*_ = *O*_*i*_ – Ō. The percent error (*E*) was also calculated to compute the error between observed and simulated values using Eq. 5.

5 $$\mathrm{Error}\left(E\right)=\left[\frac{\left({O}_{i}-{P}_{i}\right)}{{O}_{i}}\right]\times 100$$

where *n* = number of observations, *P*_*i*_ and *O*_*i*_ are predicted and observed values, respectively.

### Measurements of soil water content (SWC)

The soil water content was measured before irrigation until harvest for all treatments by adopting gravimetric sampling in 0–0.30-m depth while for the rest of soil depths from 0.30 to 1.20 m, a neutron probe (503 DR Hydroprobe, CPN International, Inc., CA. USA) was used. The aluminum access tubes were installed at 1.20-m depth in the center of each sub-plot. The neutron probe was calibrated against the soil water content determined gravimetrically. The surface soil layer (0–30 cm) was sampled gravimetrically. The four replications were taken per treatment until harvest in both growing seasons. The measured values of soil water before sowing were used as input into the model.

### Soil water balance and evapotranspiration (ET)

Accurate estimation of the soil water balance is important for determining the availability of water resources and the optimal management in agriculture (Porporato et al. 2004). In the current study, the soil water balance and ET were calculated using CSM-CROPGRO model in DSSAT. The CROPGRO-Peanut simulation model uses the Ritchie method to calculate evapotranspiration, which is used to calculate water balance in the soil. The model is based on works by Jones and Ritchie (1990) and consists of estimating crop evapotranspiration in mm day^−1^, calculating water evaporation in the soil and transpiration independently. The daily soil water balance in DSSAT is based on the one-dimensional ‘‘tipping bucket’’ approach described by Ritchie (1998).

The measured ET value was calculated using the measured SWC by a water balance method described by Allen et al. (1998). Simulated ET value was estimated with the one-dimensional water balance method described by Allen et al. (1998). The equation can be written as follows:

6 $$I+R=E+T+Dp+Rf\pm\Delta SWC$$

where *I* is the amount of irrigation water (mm); *R* is the rainfall (mm); *E* is evaporation (mm); *T* is transpiration (mm); *ET* is evapotranspiration (mm); Δ*SWC* is the change in the soil water content in the 90- cm soil profiles (mm); *Dp* is the deep percolation beyond the root zone (mm), and *Rf* is the amount of runoff (mm). The irrigation and rainfall were inputs from crop management and weather data, while other components of water balance were simulated by the model. For the seasonal water balance, the daily components were summed from sowing to harvesting in both years.

### Water productivity (WP) and irrigation water productivity (IWP)

Measured WP and IWP values were calculated as observed peanut yield divided by total measured ET and seasonal irrigation during the growing seasons. Simulated water productivity (WP) was estimated as the ratio between simulated yields and simulated seasonal ET calculated by the CROPGRO-Peanut model using Penman Monteith method (Allen et al. 1998). Simulated irrigation water productivity (IWP) was calculated as simulated peanut yield divided by total seasonal irrigation amount during the growing season, respectively (Soler et al. 2013).

## Results and discussion

### Results

#### Effect of irrigation frequencies (IF) and water level (WL) on growth and yield of peanut

The highest peanut yield (5300 kg ha^−1^) was recorded in IF_2_WL_1.25_, followed by IF_1_WL_1.25_ and IF_2_WL_1.0_ (4910 and 4670 kg ha^−1^) was observed in 2014. The lowest peanut yield was obtained from the IF_3_WL_0.50_ (2700 kg ha^−1^) in 2015. While a maximum peanut yield of 4420 kg ha^−1^ was obtained from the IF_2_WL_1.25_, followed by IF_1_WL_125_ and IF_2_WL_1.0_ with peanut yields of 4330 and 4160 kg ha^−1^, respectively in 2015. Minimum peanut yield was acquired from the IF_3_WL_50_ treatment with 1960 kg ha^−1^ in 2015.

#### Genetic coefficients estimation of CROPGRO-Peanut model

The calibrated genetic coefficients of NC-7 derived from the CROPGRO-Peanut model are presented in Table 1. Initially, coefficient EMFL and SDPM were estimated for the close fit of anthesis and maturity as followed by the approach Rahman et al. (2019). The time taken from emergence to flowering (EMFL) was 24 days, while the time acquired from first seed to physiological maturity was 84 days (Table 1). In the next step, the biomass was optimized by adjusting the LFMAX, which showed the value of 1.45 mg CO_2_/m^2^-s. Then the SLAVR was adjusted to correct the LAI peak values. Similarly, the coefficients FLSH, FLSD, and PODUR were tuned to correct the time for pod initiation and dry weight. The values of WTPSD and SFDUR were calibrated to adjust the size of the seed and shelling %. Lastly, the value of XFRT (0.94 g) was adjusted to obtain the close fit for the slop of pod or seed harvest index.

**Table 1:** Genetic coefficients of CROPGRO-Peanut model for cultivar NC-7

Coefficient	Description	Values	Unit
CSDL	Critical short-day length below which reproductive	10.84	hour
PPSEN	Slope of the relative response of envelopment to photoperiod with time	0.00	l/hour
EMFL	Time between plant emergence and flower appearance (R1)	24.3	photothermal days
FLSH	Time between first flower and first pod (R3)	8.0	photothermal days
FL-SD	Time between first flower and first seed (R5)	23.4	photothermal days
SD-PM	Time between first seed (R5) and physiological maturity (R7)	84.5	photothermal days
FL-LF	Time between first flower (R1) and end of leaf expansion	88.0	photothermal days
LFMAX	Maximum leaf photosynthesis rate at 30 C, 350 ppm CO_2_, and high light	1.45	mg CO_2_/m^2^-s
SLAVR	Specific leaf area of cultivar under standard growth conditions	270	cm^2^/g
SIZLF	Maximum size of full leaf (three leaflets)	20.0	cm^2^
XFRT	Maximum fraction of daily growth that is partitioned to seed + shell	0.94	g
WTPSD	Maximum weight per seed	1.0	g
SFDUR	Seed filling duration for pod cohort at standard growth conditions	38.0	photothermal days
SDPDV	Average seed per pod under standard growing conditions	1.65	(#/pod
PODUR	Time required for cultivar to reach final pod load under optimal conditions	30.0	photothermal days
THRSH	The maximum ratio of [seed/(seed + shell)] at maturity	75.0	%
SDPRO	Fraction protein in seeds	0.270	g(protein)/g(seed)
SDLIP	Fraction oil in seeds	0.510	g(oil)/g(seed)

#### Calibration and evaluation of CROPGRO-Peanut model for phenology

The model error (%) of different physical parameters for three irrigation frequencies and four irrigation levels is presented in Table 2. The values of simulated and observed anthesis day, maturity day, seed, and biological yield at harvest (kg ha^−1^) showed reasonable agreement during the 2014 growing season. Table 2 presents the goodness of fit parameters for seed yield and aboveground dry matter. The model showed quite satisfactory results for grain yield and biological yield of peanut in both years.

**Table 2:** Calibration results of CROPGRO-Peanut model using the data set from the three irrigation frequencies and four irrigation levels during 2014 and 2015 season

Parameters	Days to anthesis		Days to maturity		Seed yield (kg ha^−1^)		Biological yield (kg ha^−1^)	
	Error, %		Error, %		Error, %		Error, %	
	2014	2015	2014	2015	2014	2015	2014	2015
IF_1_WL_0.50_	− 3.92	− 11.7	− 1.95	− 5.44	1.52	1.69	− 6.38	17.35
IF_1_WL_0.75_	0.00	− 7.55	− 2.58	− 6.04	− 14.32	− 15.69	− 7.49	0.32
IF_1_WL_1.0_	5.36	1.72	3.05	− 1.92	3.67	− 6.63	0.94	− 12.47
IF_1_WL_1.25_	5.36	1.72	3.05	− 2.56	10.41	− 8.18	4.13	− 10.64
IF_2_WL_0.50_	0.00	− 14.0	− 1.28	− 6.90	5.37	4.01	− 5.04	15.48
IF_2_WL_0.75_	1.85	− 5.56	− 1.27	− 5.41	− 4.87	7.93	− 2.99	8.85
IF_2_WL_1.0_	7.02	1.72	3.64	− 0.64	9.40	− 8.34	− 4.66	− 6.68
IF_2_WL_1.25_	7.02	1.72	3.64	− 1.27	21.25	− 11.58	0.35	− 12.64
IF_3_WL_0.50_	− 8.16	− 18.7	− 0.65	− 5.48	3.30	− 1.73	7.36	33.22
IF_3_WL_0.75_	− 6.00	− 16.3	− 2.60	− 5.44	− 13.58	− 13.21	0.96	16.49
IF_3_WL_1.0_	0.00	− 7.55	0.63	− 1.29	− 12.11	− 9.78	− 6.59	− 2.44
IF_3_WL_1.25_	0.00	− 7.55	0.63	− 1.29	1.67	− 8.04	− 1.53	− 9.84
RMSE	2.58 days	4.92 days	3.81 days	6.29 days	462.50 kg ha^−1^	323.9 kg ha^−1^	533.5 kg ha^−1^	1516.1 kg ha^−1^
RMSE_*n*_, %	4.82	9.18	2.39	4.15	11.39	9.50	4.61	14.15
d index	0.238	0.459	0.523	0.612	0.859	0.960	0.823	0.648

The observed anthesis dates varied between 48 and 58 days depending on the irrigation interval and irrigation level in 2014 and 2015 but the model simulated the same days at all treatments (Table 2). However, the difference was 2–4 days between observed and simulated anthesis days in both years while the simulation for days to anthesis in 2015 was poor (1–9 days difference) for studied treatments (different irrigation frequencies and irrigation levels). Predictions of the maturity dates were reasonably accurate (1–6 days difference between observed and simulated in 2014) for the peanut cultivar NC-7 at all irrigation levels in 2014 and 2015. However, predictions for maturity dates under the different irrigation frequencies and irrigation levels in 2015 were rather poor, with the differences being 1–10 days. There was a 3.81- and 6.29-day difference in the entire cycle than observed days to maturity (Table 2). The IF_3_WL_0.50_ treatment reached earlier harvest maturity (153 and 146 days after sowing) in both years, while the IF_2_WL_1.25_ treatment reached harvest maturity at 165 and 157 days after sowing. The simulated days to physiological maturity ranged from 153 and 154 days for the IF_3_WL_0.50_ treatment to 159 for the IF_2_WL_1.25_ treatment in both years. The model simulated well the grain yield with RMSE values of 462 kg ha^−1^ and *d*-index of 0.85 in 2014, while low RMSE of 324 kg ha^−1^ and *d*-index of 0.95 was recorded in 2015 (Table 2). Zhao et al. (2019) evaluated the capability of the model for sowing dates and seeding rate to optimize sowing dates, seeding rate, and irrigation regimes. The results indicate that the model performed reasonably well in predicting the emergence and the majority of the growing season.

#### Leaf area index during calibration and evaluation of CROPGRO-Peanut model

**Fig. 2:**
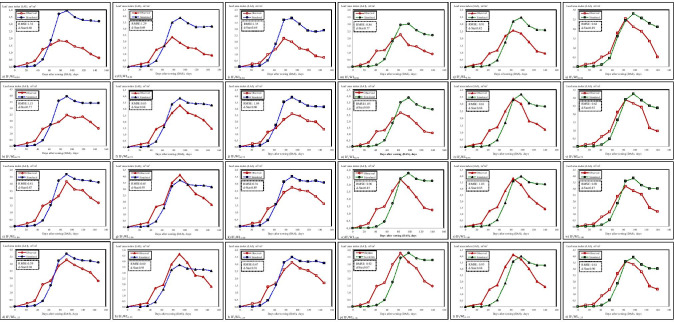
**a**–**x**. Temporal variation of observed and simulated peanut LAI values at all irrigation levels in 2014 (**a**–**l**) and 2015 (**m**–**x**)

The values of observed and simulated temporal variation in the peanut leaf area index (LAI) for 2014 are presented in Fig. 2a–l at different irrigation intervals and water levels. The RMSE values for LAI values in the IF_1_ irrigation interval range between 0.59 and 1.32, while the standard deviation (*d*-stat) values are between 0.69 and 0.94. Generally predicted values in IF_1_ irrigation interval were estimated higher than measured values. RMSE values for IF_2_ irrigation interval in 2014 ranged between 0.60 and 1.29, while *d*-stat values were between 0.69 and 0.95 (Fig. 2e–h). RMSE values for IF_3_ irrigation interval in 2014 ranged between 0.67 and 1.35, while *d*-stat values were ranged between 0.64 and 0.91. Generally, estimated values in IF_3_ irrigation interval were estimated higher than measured values (Fig. 2i–l).

The observed and simulated LAI values for 2015 are presented in Fig. 2m–x at different irrigation intervals and water levels. RMSE values for LAI values in IF_1_ irrigation interval vary between 0.86 and 1.05, while standard deviation (*d*-stat) values are between 0.77 and 0.87 (Fig. 2m–p). In 2015, the RMSE values in the IF_2_ irrigation interval varied between 0.81 and 1.03, while the standard deviation values were between 0.82 and 0.88 (Fig. 2q–t). In 2015, the RMSE values for LAI values in the IF_3_ irrigation range ranged between 0.64 and 0.89, while the standard deviation (*d*-stat) values were between 0.87 and 0.92 (Fig. 2u–x). Generally, the model performed reasonably well for LAI in both years either irrigation intervals and water levels, showing the model’s ability to predict the LAI and canopy-related attributes.

#### Modeling water productivity (WP), irrigation water productivity (IWP), and soil water content (SWC)

The measured water productivity (WP) during the 2014 cropping season ranged between 0.39 and 0.63 kg m^−3^ in the treatments, while simulated WP values ranged between 0.43 and 0.59 kg m^−3^, with 0.10 kg m^−3^ RMSE, whereas these WP values ranged from 0.32 to 0.57 kg m^−3^ with an RMSE value of 0.15 kg m^−3^ during model evaluation (2015) (Table 3). The RMSE_*n*_ was 19.8% in 2014 and 34.1% in 2015 growing seasons, showing reasonable to satisfactorily performance for WP in peanut crop under Mediterranean climatic conditions. The water productivity in the CROPGRO model under both experimental years was higher than observed. There was a positive relation between cumulative irrigation and simulated WP; as the irrigation amounts increased, the values for simulated WP increased.

**Table 3:** The observed and simulated WP and IWP of peanut in different treatments in 2014, 2015

Treatments	2014			2015		
	Observed WP, kg m^−3^	Simulated WP, kg m^−3^	Error, %	Observed WP, kg m^−3^	Simulated WP, kg m^−3^	Error, %
WP						
IF_1_WL_0.50_	0.55	0.43	21.0	0.49	0.42	14.1
IF_1_WL_0.75_	0.47	0.55	− 16.2	0.51	0.57	− 10.9
IF_1_WL_1.0_	0.47	0.55	− 18.4	0.46	0.58	− 25.6
IF_1_WL_1.25_	0.48	0.55	− 16.3	0.42	0.63	− 49.7
IF_2_WL_0.50_	0.61	0.46	24.4	0.57	0.44	22.6
IF_2_WL_0.75_	0.53	0.57	− 6.9	0.52	0.50	3.8
IF_2_WL_1.0_	0.52	0.55	− 4.8	0.48	0.64	− 33.9
IF_2_WL_1.25_	0.54	0.54	1.1	0.49	0.69	− 41.2
IF_3_WL_0.50_	0.45	0.39	14.6	0.33	0.36	− 8.1
IF_3_WL_0.75_	0.39	0.53	− 33.7	0.32	0.45	− 41.0
IF_3_WL_1.0_	0.45	0.59	− 31.1	0.36	0.55	− 53.4
IF_3_WL_1.25_	0.46	0.58	− 26.1	0.34	0.60	− 77.2
	RMSE: 0.10	RMSE_*n*_: 19.8		RMSE: 0.15	RMSE_*n*_: 34.1	
IWP						
IF_1_WL_0.50_	0.81	0.80	1.5	0.65	0.64	2.2
IF_1_WL_0.75_	0.62	0.71	− 14.3	0.54	0.62	− 15.6
IF_1_WL_1.0_	0.57	0.55	3.7	0.46	0.49	− 7.5
IF_1_WL_1.25_	0.49	0.44	10.4	0.41	0.44	− 7.9
IF_2_WL_0.50_	0.89	0.85	5.4	0.71	0.68	4.4
IF_2_WL_0.75_	0.69	0.72	− 4.9	0.59	0.54	8.1
IF_2_WL_1.0_	0.59	0.53	9.4	0.52	0.56	− 8.1
IF_2_WL_1.25_	0.53	0.42	21.2	0.44	0.50	− 12.5
IF_3_WL_0.50_	0.67	0.64	3.3	0.48	0.49	− 1.1
IF_3_WL_0.75_	0.56	0.63	− 13.6	0.40	0.45	− 12.5
IF_3_WL_1.0_	0.49	0.55	− 12.1	0.41	0.45	− 9.2
IF_3_WL_1.25_	0.45	0.44	1.7	0.37	0.40	− 6.8
	RMSE: 0.06	RMSE_*n*_: 9.3		RMSE: 0.04	RMSE_*n*_: 8.6	

Table 3 compares simulated with measured irrigation water productivity value of peanut across all calibration and evaluation data. The measured IWP during the 2014 cropping season ranged between 0.45 and 0.89 kg m^−3^ among all treatments, while simulated values ranged between 0.42 and 0.85 kg m^−3^, with the RMSE value 0.06 kg m^−3^, whereas these IWP ranged from 0.40 to 0.68 kg m^−3^ with an RMSE value of 0.04 kg m^−3^ during the model evaluation (2015). The model performed reasonably well with RMSE_*n*_ of 9.3% in 2014 and 8.6% in 2015 growing seasons.

**Fig. 3:**
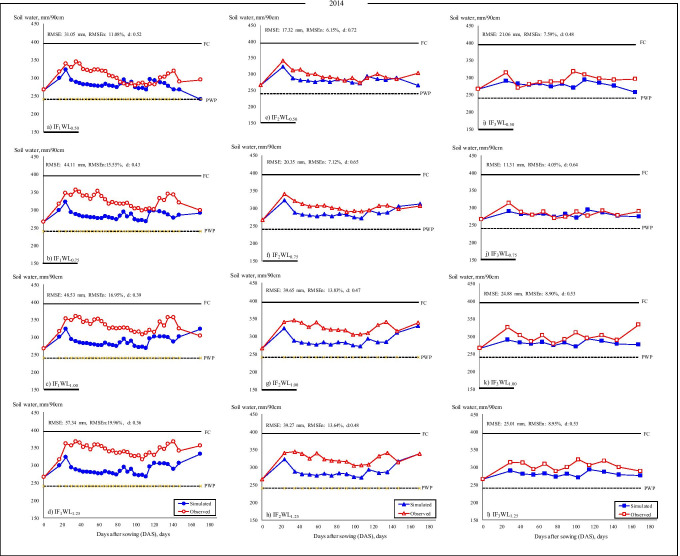
**a**–**l**. Variation of simulated and observed soil water for peanut in IF_1_ (**a**–**d**), IF_2_ (**e**–**h**), and IF_3_ (**i**–**l**) treatments in 2014

**Fig. 4:**
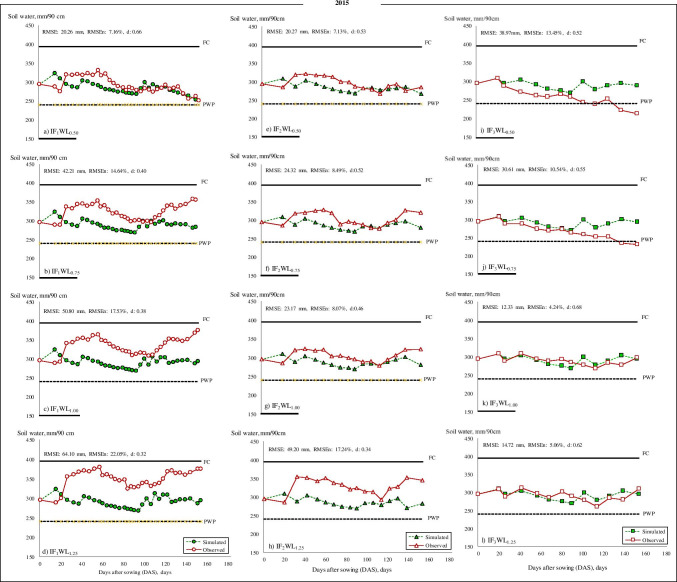
**a**–**l** Variation of simulated and observed soil water for peanut in IF_1_ (**a**–**d**), IF_2_ (**e**–**h**), and IF_3_ (**i**–**l**) treatments in 2015

The variations in measured and estimated SWC during 2014 and 2015 for each IF are shown in Figs. 3a–l and 4a–l, respectively while irrigation treatments were started on June 6, 2014, and May 23, 2015. The SWC ranged between the field capacity (FC) (395 mm) and permanent wilting point (PWP) (240 mm) for both seasons. The significant differences in the SWC among treatments from crop establishment to harvesting in both years were caused by the WL and the uptake of root water. Although deficit irrigation treatments (WL_0.50_ and WL_0.75_) received an equal volume of water under different irrigation intervals (IF_1_, IF_2,_ and IF_3_) along with the experiment, both treatments had different water uptake patterns. In the high (IF_1_) and medium (IF_2_) irrigation frequencies, SWC kept greater as compared to lower frequency (IF_3_) in both years. The available SWC in the IF_1_WL_1.0_ and IF_1_WL_1.25_ plots of the higher irrigation interval, and IF_2_WL_1.0_ and IF_2_WL_1.25_ treatment plots of the IF_2_ irrigation interval remained above 50% in most of the peanut growth stages during 2014 and 2015. Therefore, the IF_2_WL_1.0_ and IF_2_WL_1.25_ treatments performed an appropriate soil water condition for peanut. Moreover, almost in all irrigation water levels for IF_3_, the available water fell below 50% in both study years.

Various statistical analysis methods, including RMSE, RMSE_*n*_, and absolute error (*E*), was used as the evaluation indices for model calibration and evaluation to verify the reliability of the results and the goodness of fit indicators relative to SWC curves (Table 4, Fig. 3a–l, 4a–l). Simulation results indicated good agreement between measured and simulated SWC with low RMSE_*n*_ values (4.0 to 16.8% in 2014 and 4.3 to 18.2% in 2015) and lower error (*E*) values for SWC (1 to 19% in 2014 and from 0 to 20% in 2015 growing season) (Table 4). The RMSE values increased generally depending on the increasing amount of irrigation water in each irrigation interval in both years. The results showed that the model performed reasonably well in predicting the soil water content under different irrigation intervals and irrigation water levels. The high RMSE, RMSE_*n*_, and *E* values were found in WL_1.0_ and WL_1.25_ treatments with each irrigation interval in both years (Table 4).

**Table 4:** Goodness of fit indicators relative to model prediction of available soil–water (ASW) for 2014 and 2015 growing seasons of peanut for all treatments

Treatments	2014				2015			
	RMSE, mm	RMSE_*n*_, %	*d*	Error, %	RMSE, mm	RMSE_*n*_, %	*d*	Error, %
IF_1_WL_0.50_	31.0	11.1	0.52	8	20.2	7.2	0.66	3
IF_1_WL_0.75_	44.1	15.6	0.43	14	42.2	14.6	0.40	12
IF_1_WL_1.0_	48.5	16.9	0.39	15	50.8	17.5	0.38	15
IF_1_WL_1.25_	57.3	19.9	0.36	19	64.1	22.0	0.32	20
IF_2_WL_0.50_	17.3	6.1	0.72	4	20.3	7.1	0.53	4
IF_2_WL_0.75_	20.3	7.1	0.65	6	24.3	8.5	0.52	6
IF_2_WL_1.0_	39.7	13.8	0.47	12	23.3	8.1	0.46	6
IF_2_WL_1.25_	39.2	13.6	0.48	12	49.2	17.2	0.34	15
IF_3_WL_0.50_	21.1	7.5	0.48	5	38.9	13.5	0.52	10
IF_3_WL_0.75_	11.3	4.0	0.64	1	30.6	10.5	0.55	7
IF_3_WL_1.0_	24.9	8.9	0.53	7	12.3	4.2	0.68	0
IF_3_WL_1.25_	25.0	8.0	0.53	8	14.7	5.1	0.62	0

#### Summary of the findings

Deficit irrigation with less application frequency affects the phenology, growth, dry matter, and finally the yield. The treatments IF2WL1_.0_ and IF_2_WL_1.25_ showed an appropriate soil water condition for peanut growth and development. Crop reached anthesis and maturity earlier (153 and 146 days after sowing) in IF_3_WL_0.50_ due to water deficit conditions and lower SWC in soil profile while more duration was taken to maturity (165 and 157 days after sowing) in IF_2_WL_1.25_ treatments during both years. More crop duration and better growth conditions especially the available soil moisture promoted the LAI and dry matter production and finally the yield. The highest yield (5300 and 4420 kg ha^−1^) was recorded in IF_2_WL_1.25_, followed by IF_1_WL_1.25_ and IF_2_WL_1.0_ (4910 and 4330 kg ha^−1^ and 4670 and 4160 kg ha^−1^ in 2014 and 2015, respectively) while the lowest yield obtained (2700 and 1960 kg ha^−1^) from IF_3_WL_0.50_ during both studied years. Environmental and climatic conditions were found more promising for peanut growth in the first year than the second as there was a 17% higher yield obtained in 2014 than in 2015. Furthermore, the model calibration was found well and also performed reasonably well during evaluation for all studied parameters during both years. It showed the potential of the model to apply for further seasonal analysis and climate change impact assessment in the region for decision support for the peanut growers.

### Discussion

Peanut yield decreased with the reductions in the irrigation application water depending on the irrigation frequency, regimes, and amounts. The study results indicated the water stress gradually increases at lower frequency irrigations induced considerable effects on growth and reductions in peanut yield during both studies years. It could be due to loss of turgor due to lower availability of soil moisture than required by plants and it leads to drought conditions and further affects the photosynthesis and other physiological process and leads to lower LAI and dry matter production and yield (Wajid et al., 2013). Deficit irrigation treatments with less irrigation frequency (IF1) at critical crop growth phases leads to lower soil water availability and defiantly affects the plant growth and yield process which leads to a reduction in yield. Similar findings are also reported previously like Kheira (2009) demonstrated that water stress at pegging and pod development drastically reduced the yield; furthermore, he found that deficit irrigation and water stress conditions also affect the yield drastically (2200 and 3700 kg ha^−1^ for deficit and full irrigation levels) in Egypt. Rowland et al. (2012) reported average yields of peanut in all fifteen irrigation treatments varying between 4203 and 4147 kg ha^−1^ in 2005 and 2006 respectively, showing the fact and logic that water stress affects peanut growth and yield.

The model predicted phenology (3–6 day difference between observed and simulated) at various irrigation frequencies. The variation in results could be due to structural simplification of biophysical processes in the model, uncertain model inputs such as errors in weather data, and uncertainty in model parameters (Rahman et al. 2018). However, Dangthaisong et al. (2006) reported that predictions of the maturity dates were reasonably accurate for the peanut cultivar Tainan 9 at different water regimes in 2004 and at full irrigation in 2005, where the difference only ranged 0–4 days between observed and predicted. However, predictions of the maturity dates of Tainan 9 under the two water stress levels in 2005 were rather poor, with the differences being 6–7 days. Also, for the cultivar KK 60–3, the prediction of the maturity date at full irrigation in 2004 was rather poor (8 days difference) while predictions of the maturity dates at the two water stress levels were reasonably good (2–4 day differences).

Parmar et al. (2013) tested the CROPGRO-Peanut V4.5 model in 3 peanut varieties in the Gujarat region of India on different sowing dates. The model underestimated LAI values for each variety. Dugan et al. (2011) evaluated the performance of the CROPGRO-Peanut model in Ghana by simulating the response of two peanut varieties to sowing dates and sowing densities (9 and 17 plants). The changes in the leaf area index (LAI) in the model were significantly compatible with the observed values (*r*^2^ = 0.81). The study results indicated that the model simulated the temporal LAI well with lower RMSEs values (Fig. 2a–x). Similar was reported by Zhao et al. (2019) that the model performed satisfactorily in simulating LAI of peanut under different irrigation regimes and management conditions. Furthermore, Haro et al. (2008) also found the values of 3.9 for maximum LAI for peanut grown under water stress conditions and 6.2 for fully irrigated plots in Argentina. Patel and Golakia (1988) showed that continuous water deficit resulted in fewer and smaller leaves in a study conducted in India which lead to effects on growth, dry matter production, and yield. Maximum seasonal LAI for peanut tends to be greater than for most crops (Kiniry et al. 2005), with reported values for maximum LAI ranging from 3 (Gardner and Auma 1989) to greater than 8 (Chapman et al. 1993). Soler et al. (2013) reported that the observed maximum LAI ranged from 1.2 for the 30% available soil water (AWC) treatment to 6.3 for the 90% of AWC treatment.

The simulation of WP by the model was found reasonably well so the growth, development, and yield parameters were simulated in a good range (Table 3). Our results are in agreement with previous studies based on observed data for calculating WP and simulated results by Soler et al. (2013). In another study in Egypt conducted on sandy soil and under drip irrigation, reported that the peanut yield WP increased with increasing irrigation water quantity (El-Boraie et al. 2009). In water stress conditions, WP was reduced in different peanut varieties grown in Argentina (Collino et al. 2000). Bandyopadhyay et al. (2005) found that WP values ranged from 0.48 to 0.60 kg m^−3^ for peanut grown during two cropping seasons in India. Aydinsakir et al. (2016) reported that the WP varied from 0.5 to 0.75 kg m^−3^ and 0.5 to 0.8 kg m^−3^ in 2013 and 2014, respectively. Kheira (2009) reported WP values for deficit irrigated peanut in Egypt varying between 0.45 and 0.61 kg m^−3^. Our results are in agreement with the abovementioned study results and also showed the logical reasoning for the reduction in yield due to water deficit conditions. The simulated WP of peanut with different irrigation regimes ranged from 0.64 m^−3^ in a dry year under rainfed to 1.54 kg m^−3^ in a normal year. The WP of peanut increased with an increase in irrigation rate in dry years, but not in normal or wet years in Northern Chain Plain (Zhao et al. 2019).

It is reported that during the total growing period of peanut, an adequate water supply and comparatively moist soils, which are essential for good growth, development, and high yields, are required (Rao et al. 1988; Reddy and Reddy 1993). However, the flowering and fruit filling periods of peanut are more sensitive to water stress than early vegetative and late-ripening periods (Howell et al. 1980; Jain et al. 1997). The findings of related studies showed that less than 40–50% of depletion in soil water can be acceptable for higher yields (Doorenbos and Kassam 1986). Results show that the model captures SWC seasonal variations reasonably well in lower irrigation frequencies (IF_2_ and IF_3_) than higher irrigation frequency (IF_1_), and more precisely when SWC is permanently low in treatments. The observed model weaknesses appear to come mainly from SWC overestimation, all over the treatments in 2014. While the model generally gives similar results to the first experimental year, it tends to underestimate SWC late in the season with some treatments, including deficit irrigated treatments (IF_3_WL_0.50_ and IF_3_WL_0.75_). The distribution among the data shows that the observed SWC is well represented as a result of simulated values and statistical evaluations. It is concluded that CSM-CROPGRO-Peanut model is a useful tool for irrigation water management in Eastern Mediterranean region of Turkey and further it can be used for decision-making related to crop management and irrigation water application to improve resource use efficiency.

## Conclusions

The CROPGRO-Peanut model simulated the phenology, growth (LAI, dry matter production), and grain yield reasonably well with low RMSE during calibration evaluation for irrigation frequencies and water levels. The simulated WP showed a positive relation between cumulative irrigation with lower RMSE_*n*_ during both growing seasons. The IWP values decreased with increasing IF with the same WL values. Thus, it is not suggested to use IF_1_ and IF_3_ while IF_2_ can also be adopted by the farmers for drip-irrigated peanut growing in the East Mediterranean part of Turkey. Furthermore, simulation results indicated good agreement between measured and simulated SWC with low RMSE_*n*_ values (4.0 to 16.8% in 2014 and 4.3 to 18.2% in 2015) and low error (*E*) values for SWC ranged from 1 to 19% in 2014 and from 0 to 20% in 2015 growing season. IF_2_WL_1.25_ produced maximum peanut yield during both study years, followed by IF_2_WL_1.0_ while lower yield was obtained with water stress conditions in IF_3_WL_0.50_. It may be recommended that farmers may use IF_2_ (50 mm of cumulative pan evaporation) with water levels of WL_1.25_ (125 of CPE) and WL_1.0_ (100 of CPE) to achieve a good yield and save irrigation water. However, furthermore, we suggest that optimum irrigation management practices should be identified from long-term simulations for decision-making by the farmers under contrasting climatic conditions.

## Data Availability

The datasets and codes used and/or analyzed during the current study are available from the corresponding author on reasonable request.
